# Fluid requirement in adult dengue haemorrhagic fever patients during the critical phase of the illness: an observational study

**DOI:** 10.1186/s12879-021-05971-6

**Published:** 2021-03-20

**Authors:** PMW Madanayake, AEU Jayawardena, S L Wijekoon, N Perera, JKP Wanigasuriya

**Affiliations:** 1 Colombo South Teaching Hospital, Kalubowila, Sri Lanka; 2 Faculty of Medical Sciences, University of Sri Jayewardenepura, Nugegoda, Sri Lanka

**Keywords:** Dengue haemorrhagic fever, Dengue fever, Fluid requirement, Critical phase, Fluid leakage, Fluid overload

## Abstract

**Background:**

Dengue fever prevalence is rising globally and it causes significant morbidity and mortality. Fluid extravasation during the critical phase of dengue haemorrhagic fever (DHF) leads to shock, multi-organ failure and death if not resuscitated appropriately with fluids. The mainstay of management is judicious fluid replacement using a guideline based, calculated fluid quota of maintenance (M) fluid plus 5% deficit (M +  5% deficit) to prevent organ hypoperfusion.

**Methods:**

We conducted an observational follow-up study in Sri Lanka from January–July 2017 to identify the fluid requirements of DHF patients and to identify whether features of fluid overload are present in patients who exceeded the fluid quota. Patients who developed DHF following admission to the place of study, were recruited and the amount of fluid received during the critical phase was documented.

**Results:**

A total of 115 DHF patients with a mean age of 30.3 (SD 12.2) years were recruited to the study. There were 65 (56.5%) males and the mean fluid requirement was 5279.7 ml (SD 735) over the 48 h. Majority of the study participants (*n* = 80, 69.6%) received fluid in excess of the recommended maintenance + 5% deficit and this group had higher body mass index (22.75 vs 20.76, p0.03) and a lower white cell count at the onset of the critical phase (3.22 × 10^3^ vs 4.78 × 10^3^, *p* < 0.001). The highest fluid requirement was seen within the first 12 and 24 h of the critical phase in patients requiring fluid M +  5%–7.5% deficit and ≥ M +  7.5% deficit respectively. Patients exceeding M + 5% deficit had narrow pulse pressure and hypotension compared to the rest. DHF grades III and IV were seen exclusively in patients exceeding the fluid quota indicating higher amount of fluid was given for resuscitation. Fluid overload was detected in 14 (12.1%) patients and diuretic therapy was required in 6 (5.2%) patients.

**Conclusions:**

The majority of patients received fluid in excess of the recommended quota and this group represents patients with narrow pulse pressure and hypotension. Although, fluid overload was infrequent in the study population, clinicians should be cautious when administering fluid in excess of M +  7.5% deficit.

**Supplementary Information:**

The online version contains supplementary material available at 10.1186/s12879-021-05971-6.

## Background

The incidence of dengue fever has increased dramatically in the tropical and subtropical regions of the world during the recent decades with an estimated 390 million infections occurring per year, spanning across 128 countries [1, 2]. Dengue is endemic in Sri Lanka and it is a major public health problem due to associated morbidity and mortality. The first serologically confirmed dengue infected patient in Sri Lanka was identified in 1962 and the first outbreak was recorded in 1965 [3]. Over the years, dengue has caused several major outbreaks in Sri Lanka and the largest epidemic was in 2017 with 110,327 reported cases and 301 deaths during the first 7 months [4]. Surveillance data revealed that 16.9% of reported cases of dengue viral infection in the first quarter of 2017 were classified as Dengue haemorrhagic fever (DHF) [5].

Dengue infection is caused by one of the four distinct dengue virus serotypes, DENV 1–4. Many patients infected with dengue virus remain asymptomatic while symptomatic infection ranges from undifferentiated fever, dengue fever (DF), dengue haemorrhagic fever (DHF) and dengue shock syndrome (DSS) [6]. Hallmark of DHF is the plasma leakage which is transient lasting for 24–48 h [7]. Plasma leakage may result in reduced intravascular volume leading to organ hypoperfusion and multi-organ failure if not properly treated. Once fluid leakage cease at the end of 48 h, patient’s symptoms resolve and extravasated fluid is reabsorbed into the circulation. According to the World Health Organization (WHO) guidelines (revised and expanded edition 2011), four criteria are required to meet a case definition of DHF; fever, hemorrhagic tendency, thrombocytopenia (platelet count < 100,000 /mm^3^), and evidence of plasma leakage as shown by the presence of pleural effusion, ascites, or an increase in packed cell volume (PCV) by 20% [6]. Due to the specificity of these symptoms and signs, diagnosis of DHF does not require laboratory evidence of dengue virus infection [8].

Early detection of dengue and proper medical care has lowered the fatality rate below 1%. In the absence of specific antiviral therapy, meticulous fluid administration is the mainstay of management of DHF and judicious fluid administration during the “critical phase” is vital in reducing mortality [6]. The current recommended approach to fluid management in DHF requires replacement of the maintenance (M) fluid and a 5% deficit by both oral and/or intravenous administration during the critical phase of 48 h [6, 9]. Oral fluids should consist of electrolyte solutions such as oral rehydration fluid, king coconut water (a variety of coconut) and other fruit juices while normal saline is recommended for intravenous administration.

The maintenance fluid required depends on the caloric expenditure of an individual. Holliday et al. in their landmark paper published in 1957, have described the 100/50/20 rule of calculating this volume based on the body weight [10]. Although these data were derived from a paediatric population, many guidelines including the National guideline for management of dengue fever [9], have adopted the above calculation to decide on the maintenance fluid. A further 5% deficit of the maintenance is added to calculate the fluid quota during the 48 h of critical phase to a maximum weight of 50 kg (supplementary Table 1). Accordingly, the total fluid requirement during the 48-h critical phase for an average adult, weighing 50 kg or more is 4600 ml. For example, a patient weighing 56 kg will have the fluid calculated for 50 kg. Therefore, patient will receive 2100 ml of fluid as maintenance ([100 × 10] + [50 × 10] + [20 × 30]) and 2500 ml (50 × 50) of fluid as the 5% deficit over the 48 h. If the body weight is less than 50 kg, the calculation should be done according to the ideal body weight or actual body weight whichever is lower. Fluid recommended for management include crystalloid solutions administered as a continuous infusion or rapid boluses and colloids such as dextran-40 in saline [6, 9]. A few randomized trials have been conducted to identify the use of intravenous fluid in the paediatric population [11, 12]. Data in adults are lacking in this regard. Blood transfusion is recommended if bleeding is suspected during fluid resuscitation [6, 9].

There are no randomized controlled trials comparing rates and amount of fluid to be given during the critical phase of the illness. The current practice guidelines are based on consensus of expert opinions, some assumptions and studies performed on paediatric populations [13]. This involves calculation of the fluid quota for the entire critical phase and administering that volume at different rates to match the rate of fluid leakage [6]. Giving fluids liberally can have negative repercussions such as fluid overload which can cause ascites, respiratory distress from massive pleural effusions and circulatory overload leading to pulmonary oedema [14]. However, it is often observed that patients are given more than the calculated fluid quota with only few developing clinically significant fluid overload in clinical practice. Aim of this study was to evaluate the total fluid received during the critical phase of DHF and to determine whether patients developed fluid overload if excess amounts of fluid was administered. In addition, the reasons for exceeding the recommended fluid quota were also identified.

## Methods

This observational follow-up study was carried out in the University Medical Unit of Colombo South Teaching Hospital, Sri Lanka from January 2017 to July 2017. Adults above the age of 18 years who had DHF according to WHO criteria and who have not received fluid therapy before recruitment were enrolled to the study (supplementary Figure 1). Confirmation of dengue virus infection was done in doubtful situations by performing dengue NS1 antigen test and/or dengue IgM by ELISA method. Patients with co-morbidities such as heart failure, chronic liver disease and chronic kidney disease which would interfere with the fluid management were excluded from the study. A sample size calculation was performed using estimates (proportion of fluid overload in DHF taken as 15%, precision 92.5%).

Clinical parameters were recorded and the PCV was performed using capillary blood on enrolment of a patient to the study. Results of the full blood count and biochemical tests performed routinely for patient management were recorded. Duration of fever, symptoms and signs present at the time of presentation was obtained from patient inquiry and case notes. The management of the critical phase was carried out by the clinical team according to the National dengue management guideline published by the Ministry of Health in collaboration with the Ceylon College of Physicians (2012) with adjustments made according to the vital parameters, urine output and the PCV. Patients were examined at the end of the critical phase (48 h later) by the research team for evidence of fluid overload. Presence of facial puffiness, shortness of breath, large pleural effusions, fine basal crepitations or moderate ascites was considered as evidence of fluid overload.

Data were entered into a MS EXCEL database and analysed using SPSS (IBM Corp. Released 2016. IBM SPSS Statistics for Windows, Version 24.0. Armonk, NY: IBM Corp.) and GraphPad Prism version 7.0.0 for Windows, GraphPad Software, San Diego, California USA, www.graphpad.com. Continuous variables were presented as mean (standard deviation) in normally distributed data and median (IQR) in skewed data. Comparisons were performed using either t-test*,* Mann-Whitney U test, ANOVA or Kruskal-Wallis test as appropriate with Bonferroni correction for multiple comparisons. Chi squared test or the fisher’s exact test was used to compare categorical variables. A subgroup analysis was performed in patients who received fluid in excess of M + 5% deficit as discussed in the results section.

## Results

A total of 115 DHF patients diagnosed according to the WHO criteria were enrolled to the study. One participant with missing data was removed from the analysis. Serological confirmation was available in 48 (41%) of patients; NS1 antigen was positive in 46 and IgM positive in 2 patients. Patients had a mean age of 30.3 (SD 12.2) years and there were 65 (56.5%) males. The day of fever on admission ranged from day 1 to day 6 and majority of patients (*n* = 108, 93.9%) were admitted to the hospital within 4 days of onset of fever. Severity of DHF in the patients revealed that 88 (76.5%) had stable DHF (DHF Grade I &II) while 25 (21.7%) had compensated shock (DHF grade III) and 2 (1.7%) patients developed DSS (DHF grade IV). There were no deaths in the study population. Fluid received by the patients ranged from 3920 ml to 9280 ml during the 48 h of the critical phase. There were 98 (85.2%) patients who received crystalloids only while 15 (13%) patients received colloids in addition to crystalloids. The crystalloid used for fluid resuscitation was 0.9% saline and the colloid used in the study was dextran-40. There were 2 (1.7%) patients who received blood transfusion during the study period. The mean fluid requirement was 5279.7 ml (SD 735) over the 48 h in the study group. There was one outlier who received 9280 ml fluid over the 48 h and this participant was removed from analysis when comparing fluid received among groups during the 48-h period to overcome the undue effect from the extreme value. Further analysis revealed that the highest fluid requirement was within the first 24 h. The mean fluid requirement over the 0–12 h was 1395.4 ml (SD 326.2) and it was 1401.2 ml (SD 424.7) over 13–24 h. The requirement was 1298.4 ml (SD 308) and 1196.5 ml (SD 185.5) in the 25–36 h and 37–48 h of the critical phase respectively.

Patients who received fluid within the recommended quota (M + 5% deficit) and patients who received fluid in excess (>M + 5% deficit) were compared (Table 1). The baseline characteristics of the two groups were similar except for the body mass index (BMI). Patients in group 2 had higher BMI and a non-significantly higher weight than patients in group 1 (Table 1).

**Table 1:** Baseline characteristics of the study population on admission

Patient characteristic	Group 1 (≤M + 5% deficit) ***n*** = 35	Group 2 (>M + 5% deficit) ***n*** = 80	***P*** value
Age^a^	30.0 (21–37)	26.0 (19.25–38.5)	0.39
Gender, n (%)			
Male	23 (65.7)	42 (52.5)	0.19
Female	12 (34.3)	38 (47.5)	
Weight (kg)^a^	55 (50–67.5)	66.5 (55–157)	0.30
Height (cm)^a^	157.5 (150–168.2)	155 (80–166)	0.58
BMI (kg/m^2^)^a^	20.76 (19.45–23.65)	22.75 (20.22–26.21)	0.03
**Day of illness on admission, n (%)**			
< 3	8 (22.9)	21 (26.25)	0.16
3–4	23 (65.7)	56 (70.0)	
≥ 5	4 (11.4)	3 (3.75)	
**Symptoms on admission, n (%)**			
Fever	35 (100)	80 (100)	–
Headache	25 (71.43)	72 (90)	0.01
Nausea	18 (51.43)	54 (67.5)	0.10
Vomiting	16 (45.71)	50 (62.5)	0.09
Abdominal pain	9 (25.71)	18 (22.5)	0.69
**Laboratory parameters**			
WBC (×10^3^)^a^	3.6 (2.64–5.76)	3.85 (2.99–4.77)	0.44
PCV	41.84 (4.85)	41.35 (4.92)	0.49
Platelet (×10^9^)^a^	103.0 (66–166)	92.0 (63.25–140.75)	0.62
SGOT^a^	109 (77–178)	98.5 (69.75–257.5)	0.89
SGPT^a^	72 (54–123)	64 (38.25–156.25)	0.73

Mean and the standard deviation or frequency (%) shown unless specified otherwise. ^a^Median (IQR) given for skewed data and the non-parametric test (Mann-Whitney U test) used for analysis

Diagnosis of DHF in the study population was established by detection of third space fluid accumulation (pleural effusion and/or ascites) either clinically or ultrasonically, by a rise in PCV and/or haemodynamic instability (Table 2). Although evidence of fluid accumulation in the pleural and/or peritoneal space was seen similarly in both groups, hypotension and narrow pulse pressure was seen exclusively in the group of patients who received fluid in excess of M + 5% deficit.

**Table 2:** Diagnosis of DHF in the study population

Parameter	Group 1 (≤M + 5% deficit) ***n*** = 35	Group 2 (>M + 5% deficit) ***n*** = 80	***P*** value
Ultrasound evidence of fluid leakage	10 (28.57)	21 (26.25)	0.80
Clinical evidence of pleural effusion	23 (65.71)	50 (62.5)	0.74
Clinical evidence of ascites	1 (2.85)	3 (3.75)	0.81
Rise in PCV (> 20% from baseline)	3 (8.57)	19 (23.75)	0.06
Narrow pulse pressure	0 (0)	10 (12.5)	0.03
Hypotension	0 (0)	1 (1.25)	0.69

All values are given as frequency (%) and significance assessed by chi-squared or Fisher’s exact test

All patients were monitored regularly and managed according to the local dengue management guideline after the onset of the critical phase. The parameters at the onset of the critical phase in the 2 groups are given in Table 3. White cell count was significantly lower in group 2 patients at the onset of the critical phase and these patients developed fluid leakage earlier (days 4–5) compared to group 1 (days 5–6). The platelet count and the PCV were not different among the two groups.

**Table 3:** Parameters of the study population at the onset of the critical phase

Parameter	Group 1 (≤M + 5% deficit) ***n*** = 35	Group 2 (>M + 5% deficit) ***n*** = 80	***P*** value
**Haematological parameters**			
WBC^a^	4.78 (3.48–6.54)	3.22 (2.48–4.54)	< 0.001
PCV	44.05 (3.92)	43.41 (4.88)	0.07
Platelets^a^	29 (12.0–49.0)	33 (19.25–54.0)	0.12
**Day of illness developing fluid leakage, n (%)**			
3	1 (2.85)	8 (10)	0.02
4	5 (14.28)	31 (38.75)	
5	14 (40)	27 (33.75)	
6	11 (31.42)	11 (13.75)	
7	3 (8.57)	3 (3.75)	
8	1 (2.85)	0 (0)	

Values are given as frequency (%) or mean (SD) unless specified. ^a^ Denotes values given as median (IQR)

**Fig. 1:**
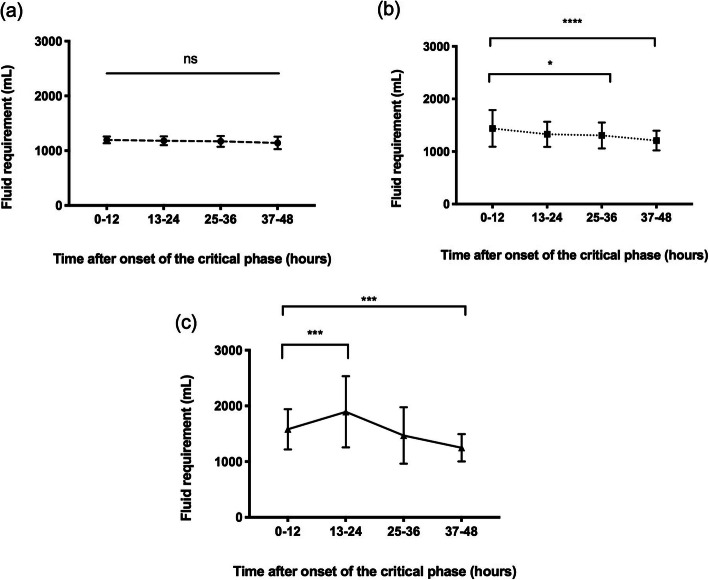
Fluid requirement over the course of 48 h of the critical phase. Figure shows the fluid requirement in the patients who received fluid (**a**) equal or less than M + 5% deficit, (**b**) M+ 5–7.5% deficit and (**c**) more than M + 7.5% deficit. Fluid requirement at 0–12 h was compared to each time point in the 3 graphs and analysed by 1-way ANOVA, *p < 0.05, ***p < 0.001, *****p < 0.0001, ns-not significant

Group 2 patients were further classified to patients who received M + 5%–7.5% deficit and patients who received fluid ≥M +  7.5% deficit. The outlier was excluded when analysing the fluid amount administered during the critical phase between groups. Analysis of the administered fluid during the critical phase (first 36 h) showed that patients requiring fluid ≥M +  7.5% received significantly higher amounts of fluid than patients in the ≤M + 5% group (*p* < 0.01) or patients who required M + 5%–7.5% deficit of fluid (*p* < 0.05) (Table 4). Figure 1 graphically shows the fluid requirement over the 48 h of the critical phase. Fluid requirement was seen to be highest during the first 12–24 h after the onset of the critical phase in patients who received fluid in excess of the recommended quota. However, the amount of fluid required in patients who received ≤M + 5% deficit were static throughout the 48 h of the critical phase (Fig. 1) indicating that they were leaking slowly.

**Table 4:** Administered fluid and the severity of the DHF in the study population

Parameter	≤M + 5% deficit n = 35	M + 5%–7.5% deficit ***n*** = 56	≥M + 7.5% deficit ***n*** = 24	P value
**Fluid administered during 0–48 h of the critical phase (ml), mean (SD)**^**a**^				
0–12	1197.4 (62.3)	1439.9 (349.3)	1596.7 (359.1)	< 0.0001
13–24	1180.4 (80.4)	1327.9 (238.6)	1831.1 (570.5)	< 0.0001
25–36	1170.1 (97.6)	1305.1 (245.6)	1391.9 (341.7)	< 0.0001
37–48	1141.3 (113.8)	1208.7 (186.4)	1238.5 (246.5)	0.01
**Category of DHF, n (%)**				
Grade I and II	35 (100)	46 (82.1)	7 (29.2)	
Grade III	0	10 (17.9)	15 (62.5)	< 0.0001
Grade IV	0	0	2 (8.3)	

^a^Analysed by kruskal-Wallis test

DHF grades III and IV were significantly higher in the group who required fluid ≥M + 7.5% deficit than the rest of the patients as shown in Table 5 (*p* < 0.0001). Patients who required fluid within the recommended quota of ≤M + 5%, did not receive isotonic saline boluses, dextran (colloid) or blood transfusion. The study population requiring fluid ≥ M + 7.5% needed saline boluses, dextran and blood transfusion more frequently than patients who received fluid M + 5%–7.5% deficit (Table 5). The reasons for giving excess fluid were identified in the study population (Table 5). The most frequent reason was identified as reduced urine output. The reasons for receiving dextran boluses were narrow pulse pressure in 5 (33%), rising PCV in 2 (13%) and reduced urine output with a rise in PCV in 8 (53%) patients.

**Table 5:** Administration of fluid boluses, blood transfusion and the reasons for giving excess fluid in the study population

Fluid type	M + 5%–7.5% ***n*** = 56	>M + 7.5% ***n*** = 24	***P*** value
Isotonic saline bolus	9 (16.07)	19 (79.16)	< 0.0001
Dextran bolus	4 (7.14)	11 (45.83)	< 0.0001
Blood transfusion	0 (0)	2 (8.3)	0.09
**Reasons for administering excess fluid identified from the patient data**			
Reduced urine output	36 (65.4)	6 (25)	
Narrow Pulse Pressure	11 (20)	15 (62.5)	
Rising PCV	5 (9.0)	1 (4.16)	
Shock	0 (0)	2 (8.33)	
Reason not identified	4 (7.14)	0 (0)	

All values are given as frequency (%). Analysed by chi-squared test or fisher’s exact test as appropriate

**Fig. 2:**
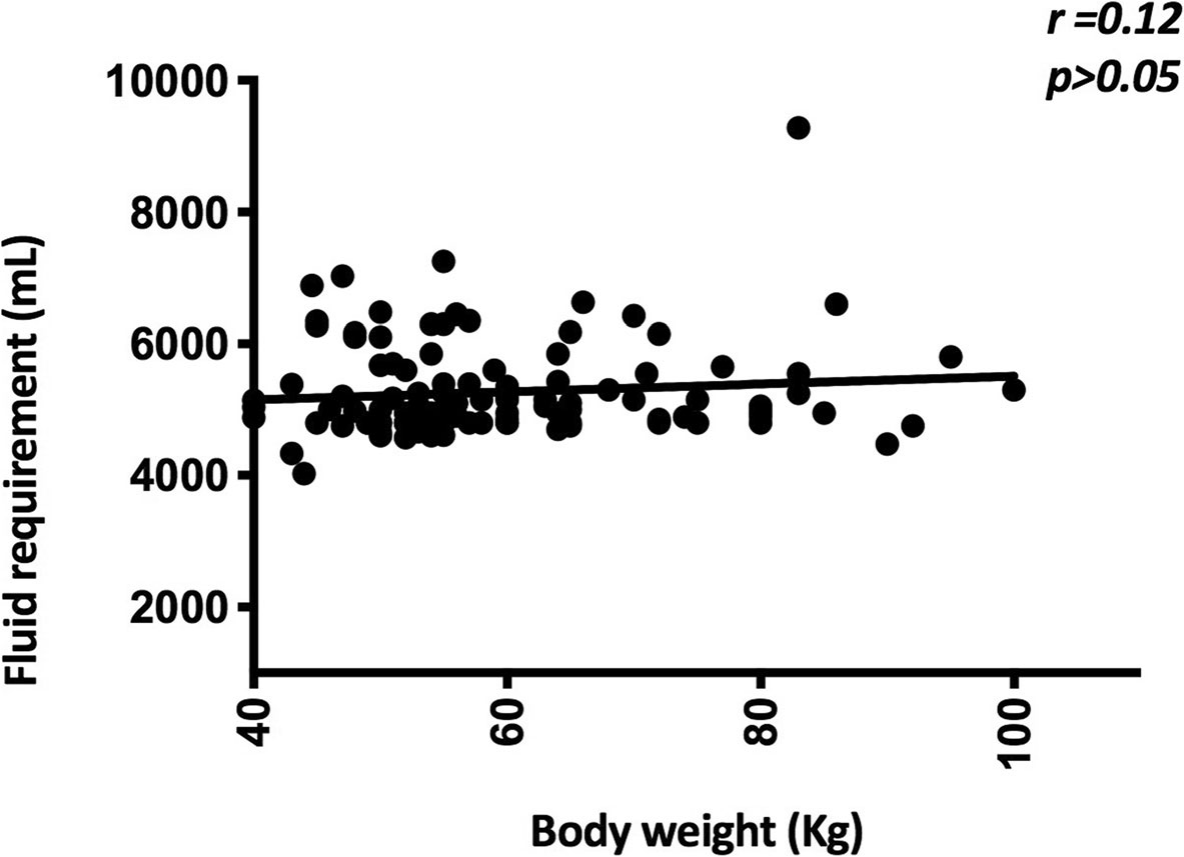
Fluid requirement of the study population based on the body weight. Fluid requirement of the patients were plotted against the body weight of the patients and analysed to identify the correlation between the variables. r- Spearman r

Fluid requirement was not seen to correlate with the body weight of the patient (Fig. 2) or the BMI of the patient. Patients exceeding body weight of 50 kg required the same amount of fluid as patients < 50 kg and patients with body weight > 80 kg required a similar amount of fluid than the rest. Fluid overload was seen in a total of 14 (12.1%) patients in the study population. Only 6 (5.2%) patients in the ≥M+ 7.5% deficit group developed moderate-severe fluid overload (Table 6). Diuretic therapy was required in 6 (5.2%) patients to relieve fluid overload symptoms and these patients received excess fluid due to narrow pulse pressure and a rise in PCV.

**Table 6:** Presence of fluid overload in the study population

Fluid overload	≤M + 5% ***n*** = 35	M+ 5% - M+ 7.5% ***n*** = 56	≥M+ 7.5% ***n*** = 24	***P*** value
None	35 (100)	54 (96.4)	12 (50)	< 0.0001
Mild	0	2 (3.6)	6 (25)	
Moderate-severe	0	0	6 (25)	

Values given as frequency (%)

## Discussion

This observational follow-up study has revealed important observations in fluid given to patients with DHF. Our cohort of patients had accumulation of fluid in the third space detected clinically or ultrasonically. However, a PCV rise above 20% of baseline was seen only in 19% of patients. In a study by Premaratna et al., only three out of 112 patients with DHF had ≥20% rise in PCV and all of them developed DHF grade III according to WHO criteria [15]. Patients who are on intravenous fluid during early phase of illness may not show the expected rise in PCV and rises above ≥20% seems to indicate high amount of fluid leakage and more severe stages of dengue. We found that 80 (69.6%) patients received more than M + 5% deficit fluid during the critical phase with only 35 (30.4%) patients being managed with fluid at or below the recommended M + 5% deficit. A study by Kularatne et al. in Sri Lanka (2015) revealed approximately 3000 ml fluid per 24 h being required in DHF patients to maintain vital parameters [16]. Fluid requirement in our patients was calculated according to the national guidelines [9] and the infusion rate of fluid was adjusted according to the patient’s clinical parameters to maintain effective circulation during the critical phase. One patient required a staggering 9280 ml fluid during the critical phase to maintain vital parameters and this patient also required saline boluses, dextran and blood transfusion and subsequently developed moderate-severe fluid overload. Patients who required fluid in excess of M + 5% deficit had a higher BMI but the amount of fluid received did not significantly correlate with the BMI or the weight. Patients requiring excess fluid had a lower mean white cell count at the onset of the critical phase and this group of patients developed evidence of fluid leakage earlier during the course of illness than the other group (day 4–5 vs day 5–6). In addition, they represented a group with more severe fluid extravasation as evidenced by haemodynamic instability and more severe disease (DHF grades III and IV) compared to patients requiring fluid within the recommended quota. There was no significant difference in other demographic parameters between the two groups or laboratory parameters such as thrombocytopaenia and elevation of liver enzymes. In addition, patients who required fluid ≥M + 7.5% deficit had severe disease than the rest and required fluid boluses (isotonic saline and dextran) and blood transfusion. Thus, our data shows that DHF represents a heterogeneous population with variable fluid leakage and patients with severe disease require fluid in excess of the currently recommended M + 5% deficit to maintain organ perfusion.

Plasma leakage in DHF starts slowly, gradually peaks and cease at the end of the critical phase. However, it is highly variable from patient to patient. In keeping with the dynamic nature of fluid leakage during the critical period, the fluid should be started at a slower rate and increased at a stepwise pattern according to the urine output, clinical parameters and the PCV. This pattern was observed in patients who required fluid in excess of M + 5% deficit and it was most marked in patients requiring fluid in excess of M + 7.5% deficit. The amount of fluid required in patients who received ≤M + 5% deficit was static throughout the 48 h of the critical phase (Fig. 1a). Fluid requirement is calculated for a maximum body weight of 50 kg irrespective of the weight of the patient. Our data showed that fluid requirement indeed did not increase in patients with body weights exceeding 50 kg and even 80 kg compared to patients with a weight less than 50 kg in keeping with the fact that total body water is similar in these patients. Reduced urine output was the commonest cause for exceeding the recommended fluid quota followed by narrow pulse pressure, rising PCV and shock. In stable patients, giving excess fluid for reduced urine output was the reason for exceeding the recommended quota. Data also revealed that some patients received fluid boluses for reduced urine output despite normal haemodynamic parameters. Giving excess fluid could have been prevented in some of these patients if more stringent criteria were adopted when administering fluid boluses. It is important to strike a balance between maintaining adequate organ perfusion and preventing fluid excess.

Contrary to the belief that exceeding the recommended fluid quota results in fluid overload, it was uncommon in our study population. Despite exceeding the recommended fluid quota in a proportion of patients, fluid overload was seen only in 14 (12.1%) patients in the study population. Mild fluid overload as evidenced by facial puffiness was observed in 2 (3.6%) patients who received M+ 5% -7.5% deficit amount of fluid and 6 (24%) who received ≥M+ 7.5% deficit amount of fluid. Moderate to severe fluid overload (moderate to severe ascites, large pleural effusion and shortness of breath) was rare and observed in 6 out of 24 patients (5.2% of the total population) who received ≥M+ 7.5% deficit amount of fluid. Diuretic therapy was required in 6 (5.2%) patients to relieve symptoms of fluid overload.

This study is a single centre experience in management of DHF. The management of DHF could vary between units and it is important to have a multicentre study to understand the patterns of fluid therapy prior to generalising the data. The observational nature of the study poses limitations on predicting causality and a prospective randomised controlled trial on different fluid regimens would be useful in this aspect to improve existing guidelines. Any observer bias that could occur due to investigators being involved in patient management was minimised by data collection and analysis done by researchers external to the treatment team. Also, dengue serology and/or NS1 antigen testing was not performed in all patients and this is a minor drawback in the study. A further limitation of the study was not excluding malaria in the study population by specific investigations. However, malaria has been eradicated from Sri Lanka and few exported cases are detected in the country.

## Conclusions

This study has shown that fluid requirement during the critical stage of DHF is highly variable and majority of patients require fluid in excess of the currently recommended M + 5% deficit to maintain the clinical parameters. Patients requiring fluid in excess of ≥M+ 7.5% deficit represented a group with more severe fluid extravasation as evidenced by haemodynamic instability and more severe disease. Clinically significant fluid overload and the need for diuretic therapy are infrequent in adult DHF patients but clinicians need to be cautious when exceeding fluid amount beyond M+ 7.5% deficit. The rate of infusion should be increased instead of giving fluid boluses in patients with reduced urine output in the absence of hypovolaemia. However, restricting the amount of fluid in fear of fluid overload may lead to impaired organ perfusion and tissue hypoxia.

## Supplementary Information

**Additional file 1: Supplementary Table 1:** Calculation of the total fluid requirement in the critical phase (48 h): M+ 5% deficit.

**Additional file 2.**

## Data Availability

The datasets supporting the conclusions made in this article are included in the results. The dataset is also available from the corresponding author on reasonable request.

## Abbreviations

DF: Dengue fever
DHF: Dengue haemorrhagic fever
DSS: Dengue shock syndrome
PCV: Packed cell volume
M: Maintenance
WHO: World Health Organization
BMI: Body mass index
