# Supplementary material for: Fluid requirement in adult dengue haemorrhagic fever patients during the critical phase of the illness: an observational study
Source: BMC Infect Dis. 2021 Mar 20;21:286. doi: 10.1186/s12879-021-05971-6 (PMC7981820; doi:10.1186/s12879-021-05971-6)
Supplement: Supplementary file 1 — Additional file 1: Supplementary Table 1: Calculation of the total fluid requirement in the critical phase (48 h): M+ 5% deficit. [file 12879_2021_5971_MOESM1_ESM.docx]

**Supplementary table 1: Calculation of the total fluid requirement in the critical phase (48 hours): M+ 5% deficit**

| **Maintenance (M)** | | **5% deficit** |
| --- | --- | --- |
| For the 1st 10 kg | 100 ml /kg | 50 ml/kg up to 50 kg |
| For the 2nd 10 kg | 50 ml/kg |  |
| From 20 kg and above up to 50 kg | 20 ml/kg |  |
