# Supplementary material for: Fluid requirement in adult dengue haemorrhagic fever patients during the critical phase of the illness: an observational study
Source: BMC Infect Dis. 2021 Mar 20;21:286. doi: 10.1186/s12879-021-05971-6 (PMC7981820; doi:10.1186/s12879-021-05971-6)
Supplement: Supplementary file 2 — Additional file 2. [file 12879_2021_5971_MOESM2_ESM.dotx]

Patients admitted with dengue fever to the place of study (Professorial medical unit at Colombo South Teaching Hospital)

Fluid therapy started prior to recruitment

Patients developing fluid leakage during ward stay

Age>18 years

- Chronic kidney disease*
- Heart failure #
- Chronic liver disease β

Excluded from study

Enrolled for the study

*Chronic kidney disease was defined as a previous diagnosis of chronic kidney disease or persistent elevation of serum creatinine with a e-GFR<60ml/min/1.73m^2^ during hospital stay

# Heart failure was defined as a previous diagnosis of heart failure and/or any participant on treatment for heart failure with a left ventricular ejection fraction <40%.

β Chronic liver disease was defined as a previous diagnosis of chronic liver disease or biochemical evidence of progressive liver failure persistent for more than 6 months.

**Supplementary figure 1: Flow chart demonstrating the recruitment and exclusion of patients in the study.**
